# Is the Utility of the GLIM Criteria Used to Diagnose Malnutrition Suitable for Bicultural Populations? Findings from Life and Living in Advanced Age Cohort Study in New Zealand (LiLACS NZ)

**DOI:** 10.1007/s12603-022-1874-9

**Published:** 2023-01-10

**Authors:** Sue O. MacDonell, S.A. Moyes, R. Teh, L. Dyall, N. Kerse, C. Wham

**Affiliations:** 1 Department of General Practice and Primary Health Care, Faculty of Medical and Health Sciences, University of Auckland, Auckland, New Zealand; 2 School of Sport, Exercise and Nutrition, Massey University, Auckland, New Zealand

**Keywords:** Advanced age, SCREEN-II, malnutrition screening, GLIM, mortality

## Abstract

**Objectives:**

To investigate associations between nutrition risk (determined by SCREEN-II) and malnutrition (diagnosed by the GLIM criteria) with five-year mortality in Māori and non-Māori of advanced age.

**Design:**

A longitudinal cohort study.

**Setting:**

Bay of Plenty and Lakes regions of New Zealand.

**Participants:**

255 Māori; 400 non-Māori octogenarians.

**Measurements:**

All participants were screened for nutrition risk using the Seniors in the Community: Risk Evaluation for Eating and Nutrition (SCREEN-II). Those at high nutrition risk (SCREEN-II score <49) had the Global Leadership Initiative in Malnutrition (GLIM) criteria applied to diagnose malnutrition or not. Demographic, physical and health characteristics were obtained by trained research nurses using a standardised questionnaire. Five-year mortality was calculated from Government data. The association of nutrition risk (SCREEN-II) and a malnutrition diagnosis (GLIM) with five-year mortality was examined using logistic regression and cox proportional hazard models of increasing complexity.

**Results:**

56% of Māori and 46% of non-Māori participants had low SCREEN-II scores indicative of nutrition risk. The prevalence of GLIM diagnosed malnutrition was lower for both Māori and non-Māori (15% and 19% of all participants). Approximately one-third of participants (37% Māori and 32% non-Māori) died within the five-year follow-up period. The odds of death for both Māori and non-Māori was significantly lower with greater SCREEN II scores (better nutrition status), (OR (95% CI); 0.58 (0.38, 0.88), P < 0.05 and 0.53 (0.38, 0.75), P < 0.001, respectively). GLIM diagnosed malnutrition was not significantly associated with five-year mortality for Māori (OR (95% CI); 0.88 (0.41, 1.91), P >0.05) but was for non-Māori. This association remained significant after adjustment for other predictors of death (OR (95% CI); 0.50 (0.29, 0.86), P< 0.05). Reduced food intake was the only GLIM criterion predictive of five-year mortality for Māori (HR (95% CI); 10.77 (4.76, 24.38), P <0.001). For non-Māori, both aetiologic and phenotypic GLIM criteria were associated with five-year mortality.

**Conclusion:**

Nutrition risk, but not malnutrition diagnosed by the GLIM criteria was significantly associated with mortality for Māori. Conversely, both nutrition risk and malnutrition were significantly associated with mortality for non-Māori. Appropriate phenotypic criteria for diverse populations are needed within the GLIM framework.

## Introduction

**M**alnutrition in older age refers to a state of undernutrition, characterised by poor appetite, unintentional weight loss and changes in body composition (1). Older adults are at increased risk of malnutrition due to altered sensory perception, reduced hunger signals, and early satiety. Comorbidities and altered psycho-social characteristics can also contribute to greater nutrition risk (2, 3). In addition, valuing and having access to traditional foods has been associated with a lower nutrition risk for older Māori (4). Nutrition risk increases with age and is a contributing factor to hospitalisation (5), and mortality in national studies of aging (5, 6, 7). There is limited information, however, regarding the prevalence and consequence of malnutrition in those of advanced age (≥ 85 years) who are the fastest growing demographic of older adults (8).

The ‘Seniors in the Community: Risk Evaluation for Eating and Nutrition' (SCREEN II) is a nutrition risk screening tool specifically designed for community living older people. It examines three domains of known risk factors: Weight Change, Dietary Intake, and Factors Affecting Intake (9). SCREEN II was initially validated among older people in Canada (10) and latterly, for octogenarians in New Zealand (11).

SCREEN II was used in the baseline assessment of the Life and Living in Advanced Age Cohort Study in New Zealand (LiLACS NZ) and identified a high prevalence of nutrition risk among Māori (49%) and non-Māori (38%) participants (3). Furthermore, low scores in the Dietary Intake domain of SCREEN II were associated with a significantly greater risk of five-year all cause hospitalisation and mortality for Māori participants. There was, however, no significant association with any SCREEN II domain for non-Māori, nor were the outcomes of overall nutrition risk investigated (7).

Malnutrition screening tools, including SCREEN II, are well established methods that identify potential nutrition risk. There remains, however, a lack of international consensus regarding the clinical criteria that are needed to obtain a diagnosis of malnutrition (12). In 2019, a collaboration of leading international nutrition support organisations known as the Global Leadership Initiative in Malnutrition (GLIM), proposed a diagnostic scheme for malnutrition aimed at addressing this lack of consensus (12). The resulting diagnostic GLIM model consists of two steps.

Firstly, a validated malnutrition screening tool is used to screen for nutrition risk. The second step confirms the diagnosis of malnutrition if there is the concurrent presence of at least one phenotypic criterion: (non-volitional weight loss OR low body mass index (BMI) OR reduced muscle mass), AND at least one aetiologic criterion (reduced food intake/assimilation OR disease burden/ inflammatory conditions) (12). The GLIM criteria are designed to provide a common basis for diagnosing malnutrition while allowing for global comparisons of malnutrition prevalence and outcomes (12). GLIM contributors note that the consensus criteria are expert opinion and recommend that validation and reliability testing is undertaken in diverse populations to assist with refinement and applicability of the criteria (13).

Preliminary work has focused on the predictive validity of the GLIM criteria, with studies to date identifying that GLIM is predictive of mortality in community-dwelling older adults. These studies, however, were conducted in cohorts of adults with an average age range of 72 to76 years (14, 15, 16) and to date, no studies have applied the GLIM criteria to adults of advanced age (≥ 80 years).

The GLIM consensus group identified alternative BMI criterion for use with older adults (BMI < 22 kg/m^2^ vs <20 kg/m^2^ for those under 70 years of age) and Asian adults (<18.5 kg/m^2^ for those under 70 years and <20 kg/m^2^ if >70 years of age) (12). However, no alternative BMI criteria have been included for population groups who have differing body composition and anthropometric profiles. Māori, the indigenous people of New Zealand, have a higher proportion of lean body mass compared to non-Māori and lower body fat percentage when the same BMI is compared with non-Māori populations (17).

While the GLIM criteria show potential as a global malnutrition definition, comprehensive investigations are needed to confirm its usefulness in advanced age and other ethnic groups. The aim of this study was to investigate potential associations of the GLIM criteria (diagnosis of malnutrition), and of the SCREEN-II (nutrition risk), with five-year mortality in Māori and non-Māori of advanced age.

## Methods

This cohort mortality estimation study extends analysis of data from the baseline assessment of Life and Living to Advanced Age: A Cohort study New Zealand (LiLACS NZ) Te Puāwaitanga o Nga Tapuwae Kia Ora Tonu (18). Māori who were aged 80–90 years and non-Māori aged 85 years and who lived in a geographically defined area of the North Island, New Zealand were invited to participate. The younger eligibility age for Māori was due to the gap in life expectancy between Māori and non-Māori (8.6 years for men and 7.9 years for women) (19). Recruitment of 421 Māori and 516 non-Māori participants occurred in 2010 with follow-up occurring annually thereafter for five years (2011 – 2015). Trained research assistants administered comprehensive sociodemographic and health questionnaires to collect all data reported here. Ethical approval was provided by the Northern X Regional Ethics Committee, NZ (NXT 09/09/088) in 2009, and all participants provided written consent.

### Sociodemographic and health data

Socioeconomic deprivation was established using the NZ Deprivation Index 2006 (NZDep(2006)) which uses the participant's home address to assign a level of deprivation based on eight dimensions: income, employment status, transport, household crowding, home ownership, education qualifications, support and communication (20). Deciles were grouped as high (deciles 1–4), moderate (deciles 5–7) and low deprivation (deciles 8–10). Smoking status was determined from participants' response to the question ‘do you smoke, or have you ever smoked cigarettes?' Responses were classified as having never smoked, current or past smokers. Self-reports of (19) chronic medical conditions were cross checked with medical records and the total number of diagnoses summed to establish the number of comorbidities (21). Disease burden was defined by the presence of malignant disease, chronic obstructive pulmonary disease, congestive heart failure and/or chronic renal disease as outlined in the GLIM guidelines (12). All prescription medications that were being taken at baseline were recorded and then tallied to be treated as a continuous variable.

### Anthropometry and Body Composition

All measures were taken in light clothing and bare feet using standardised protocols (18). Body weight was measured using the Tanita Inner Scan Body Composition Monitor, BC-545 (Tanita Corporation, Tokyo, Japan). The same scale also measured fat free mass (FFM) via bioelectrical impedance analysis (BIA). While this is not the most accurate method of body composition estimation, other more sophisticated measures, such as dual x-ray absorptiometry (DXA) were not feasible for use in the community setting. A portable seca 213 stadiometer, (seca Corporation, Hamburg, Germany) was used to measure height (m) except where participants were unable to stand. For these participants, height was estimated from the proxy measure of demi-span (22). Body mass index (BMI) and fat free mass index (FFMI) were calculated by dividing weight (kg) and FFM (kg) respectively by height (m^2^).

### Blood sampling

Fasting blood samples from consenting LiLACS NZ participants were collected at baseline by trained phlebotomists. Samples were centrifuged and stored at −80 degrees Celsius required for processing. High sensitivity C-reactive protein (hs-CRP) was measured by a laboratory that met International Accreditation New Zealand standards, using the antigen-antibody method, immunoturbidimetric reading and processed on the P module of the Roche Modular analyser (Roche Diagnostics GmbH, Mannheim, Germany).

### Screening for nutrition risk

The Seniors in the Community: Risk Evaluation for Eating and Nutrition, version II, (SCREEN II) was used to establish nutrition risk at baseline. This 14 item questionnaire scores responses to questions in three domains: weight change; factors impacting food intake (meal frequency, diet restriction, appetite, chewing and swallowing difficulties, use of meal replacements, eating alone, meal preparation and shopping difficulties); and the dietary intake domain which examines skipping meals, limiting food, appetite, fruit, vegetable, meat (and alternatives), milk and fluid intake. SCREEN II has been validated for use with non-Māori New Zealand adults of advanced age with a score ≤ 49 out of a possible 64 indicating significant nutrition risk (11).

### Malnutrition diagnosis: GLIM criteria

Participants identified as being at high nutrition risk were subsequently assessed using the GLIM criteria (12). Diagnosis of malnutrition required the presence of at least one phenotypic and one aetiologic GLIM criterion as outlined in Table 1. Non-volitional weight loss was estimated from the SCREEN II item data for participants who had lost ≥ 2.5kg and who provided a negative response to the question ‘have you been trying to change your weight in the past 6 months?' Participants who weighed ≥ 100kg needed to have lost more than 5 kg (≥5%), those who weighed less than 100kg indicated weight loss in the range of 2.5 – 5kg. Categorisation of malnutrition as moderate or severe was not examined due to limited information regarding the degree of non-volitional weight loss.

**Table 1:** Definition of GLIM criteria using LiLACS NZ data

	**GLIM Criteria**	**LiLACS NZ Definition**
Phenotypic	Non-volitional weight loss	Non-volitional weight loss over previous 6 months identified by SCREEN-II
	Low BMI	aged ≥ 70 years (all participants): BMI < 22 kg/m^2^
	Reduced muscle mass	Men: FFMI <17 kg/m^2^, ^a^ Women: FFMI <15 kg/m^2^, ^a^
Aetiologic	Reduced food intake or assimilation	Self-described ‘poor' appetite in SCREEN-II
	Disease burden/ inflammatory condition	Presence of chronic disease^b^ Elevated serum hs-CRP (> 5 mg/L)^c^

a. ESPEN recommended cut-offs 1; b presence of malignant disease, chronic obstructive pulmonary disease, congestive heart failure, chronic renal disease 12; c. for sensitivity analysis; Abbreviations: BMI, body mass index [weight (kg)/(height (m))2]; hs-CRP, high sensitivity C-reactive protein; ESPEN, European Society for Parenteral and Enteral Nutrition; FFMI, fat free mass index [fat free mass (kg)/(height (m))2]; GLIM, Global Leadership Initiative on Malnutrition; Screen-II, Seniors in the Community: Risk Evaluation for Eating and Nutrition, version II.

### Mortality outcome measure

Mortality data was accessed from New Zealand Health Information Services (NZHIS) and District Health Board data. Time to death from enrolment was used to calculate the number of days alive in the five-year follow-up period and the number of days alive and out of any hospital (Aged Residential Care and Public Hospital).

### Statistical methods

Data collected at the time of LiLACS NZ enrolment (2010) forms the baseline for this study. Mortality data was obtained annually for five years of follow-up. All statistical analysis was completed using SAS/STAT 9.4 (Copyright © 2016 Institute Inc., Cary, NC, USA). Descriptive statistics were completed for all demographic and health variables. Categorical variables are reported as frequency (percentage,%) and continuous variables are presented as means and standard deviations (SD). Regression analysis was performed separately for Māori and non-Māori cohorts to enable ethnic specific interpretation of the findings.

The association of nutrition risk (SCREEN-II) and a malnutrition diagnosis (GLIM) to mortality over five years was examined using both logistic regression and Cox proportional hazard models of increasing complexity. Models were first run with SCREEN II (nutrition risk) as the only predictor (Model A), and further adjusted for age (Māori models only), sex, deprivation, and smoking status (Model B). Age was not adjusted for in non-Māori models, as all non-Māori cohort members were the same age at enrolment. The third model was further adjusted for the number of comorbidities and medications (Model C). Each model was then re-run with the GLIM criteria (malnutrition diagnosis) replacing SCREEN II. Models that examined SCREEN-II were reported as ten points difference (rather than one point) to determine a clinically significant difference.

Death (yes/no) in the five years from baseline was the response variable in the logistic regression models. In the Cox proportional hazard models, the response variable was whether death had occurred during the five years of follow-up and, when they were last alive in those five years.

A sensitivity analysis substituting disease burden for hs-CRP as the indicative inflammatory aetiologic criterion was also performed for each model.

The association of each individual phenotypic and aetiological GLIM criterion with five-year mortality were also examined using Cox proportional hazard models constructed in the same way as the main models described above; (i.e., Model A each criterion as the only predictor; model B adjusted for age (Māori models only), sex, deprivation, and smoking status; and Model C was further adjusted for the number of comorbidities and medications).

## Results

Baseline demographic, health, and anthropometric characteristics of 655 LilACS NZ participants with complete nutrition risk data are shown in Table 2. Māori participants were on average younger than non-Māori, and there was a higher prevalence of nutrition risk in Māori participants (56% and 46% respectively). However, few participants were diagnosed as malnourished when the GLIM criteria were applied.

**Table 2:** Sociodemographic, anthropometric and health characteristics of 255 Māori and 400 non-Māori New Zealanders aged 80 years and over with complete nutrition risk data

	**n**	**Māori**	**n**	**non-Māori**
Age at baseline (years), mean (SD)		82.3 (2.6)		84.6 (0.5)
Sex, n (%)	255		400	
Men		100 (39)		188 (47)
Women		155 (61)		212 (53)
Socioeconomic Deprivation^a^, n (%)	255		400	
Deciles 1 to 4		31 (12)		100 (25)
Deciles 5 to 7		57 (22)		145 (36)
Deciles 8 to 10		167 (65)		155 (39)
Smoking, n (%)	252		400	
Non-smokers (never smoked)		110 (44)		213 (53)
Current Smokers		32 (13)		19 (5)
Past Smokers		110 (44)		168 (42)
Number of medications, mean (SD)	254	4.9 (3.3)	400	5.4 (3.5)
Elevated hs-CRP (> 5 mg/L), n (%)	188	45 (24)	339	81 (24)
Nutrition Risk (SCREEN II)	255		400	
SCREEN-II score, mean (SD)		48.0 (6.2)		49.7 (6.4)
High nutrition risk (SCREEN-II ≤ 49), n (%)		142 (56)		183 (46)
GLIM Phenotypic criteria (GLIM)				
Body weight (kg)	226	76.4 (16.7)	360	71.2 (12.5)
non-volitional weight loss, n (%)	255	15 (6)	400	23 (6)
Body Mass Index, n (%)	226			359
< 18 kg/m^2^		0 (0)		4 (1)
18-22 kg/ m^2^		17 (8)		27 (8)
22.1-25 kg/ m^2^		31 (14)		84 (23)
> 25 kg/ m^2^		178 (79)		244 (68)
Fat Free Mass (kg)	208	49.1 (10.0)	334	47.5 (9.6)
Fat free mass index (FFMI) (kg/m^2^)		18.9 (2.8)		17.8 (2.4)
Reduced muscle mass^b^, n (%)		13 (6)		46 (14)
GLIM Aetiologic criteria				
Reduced food intake, n (%)	255	1 (<1)	400	6 (2)
Number of comorbidities, mean (SD)	255	4.6 (1.0)	400	4.7 (1.0)
Disease burden, n (%)		253 (99)		399 (100)
GLIM Malnutrition^c^				
GLIM malnutrition diagnosis^d^, n (%)	142	25 (18)	183	44 (24)
Mortality	255		400	
Died in five years, n (%)		95 (37)		129 (32)
Days alive in five years of 1826 days available		1493 (545)		1558 (489)

Data presented as mean (SD) unless otherwise noted. a. NZ Deprivation Index (2006) (20); b. Low FFMI: men <17 kg/m^2^ and women <15 kg/m^2^ (1); c. Presence of 1 phenotypic and 1 aetiologic GLIM criteria; d. GLIM diagnosis made with disease burden as the inflammatory criterion; Abbreviations: ARC, aged residential care; GLIM, Global Leadership Initiative in Malnutrition; hs-CRP, high sensitivity C-reactive protein; Screen-II, Seniors in the Community: Risk Evaluation for Eating and Nutrition, version II.

There was a low prevalence of the three phenotypic criteria for both cohorts (Table 2). Few participants (6%) had experienced non-volitional weight loss, and less than 10% of both Māori and non-Māori met the GLIM criterion for low BMI (<22kg/m^2^). More non-Māori compared to Māori had reduced FFMI, although the prevalence was still low at 14%. Prevalence of the two aetiologic criteria varied. The inflammation criterion, indicated by disease burden, was the most prevalent GLIM criterion, whereas self-reported reduced food intake was virtually absent (Table 2).

Days alive accounted for an average of approximately 4.1 years of the five-year follow-up period for both Māori and non-Māori (Table 2). Approximately one-third of all participants (37% of Māori and 32% of non-Māori) died within the five-year follow-up period. Increasing age and smoking were significant risk factors of death for Māori.

### Nutrition risk as a predictor of mortality

Nutrition risk, indicated by a SCREEN-II score ≤49, was significantly associated with mortality for both Māori and non-Māori (Table 3). For every 10-point increase in SCREEN II score (better nutrition status), both Māori and non-Māori had significantly lower odds of death within five years (OR (95% CI); 0.58 (0.38, 0.88)), P < 0.05 and 0.53 (0.38, 0.75), P < 0.001, respectively. This relationship was sustained after adjustment for other covariables (OR (95% CI); 0.44 (0.27, 0.73), P < 0.01 and 0.53 (0.36, 0.76), P < 0.001 respectively) (Table 3).

**Table 3:** Logistic regression models for mortality in five years of follow-up for 142 Māori and 183 non-Māori LiLACS NZ participants identified at nutrition risk

	**SCREEN II (10 points greater)**			**GLIM malnutrition diagnosis**^**a**^**(no malnutrition vs malnutrition)**		
	**Model A Odds Ratios (CI)**	**Model B Odds Ratios (CI)**	**Model C Odds Ratios (CI)**	**Model A Odds Ratios (95% CI)**	**Model B Odds Ratios (95% CI)**	**Model C Odds Ratios (95% CI)**
Māori Older Adults						
Malnutrition indicator	0.58 (0.38, 0.88)*	0.42 (0.26, 0.69)***	0.44 (0.27, 0.73)**	0.91 (0.46, 1.82)	1.05 (0.50, 2.23)	0.88 (0.41, 1.91)
Age (one year older)		1.21 (1.09, 1.35)***	1.22 (1.09, 1.36)***		1.17 (1.06, 1.28)**	1.19 (1.07, 1.32)**
Sex (M vs F)		2.31 (1.25, 4.26)**	1.05 (0.96, 1.15)**		2.10 (1.22, 3.63)**	1.07 (0.99, 1.17)*
Deprivation^b^ (deciles 1–4 vs 8–10)		0.99 (0.40, 2.46)	2.35 (1.27, 4.37)		0.59 (0.26, 1.34)	2.16 (1.19, 3.92)
Deprivation (deciles 5–7 vs 8–10)		1.10 (0.55, 2.19)	0.96 (0.38, 2.41)		0.87 (0.46, 1.65)	0.71 (0.3, 1.70)
Smoking (Current vs Never)		4.20 (1.72, 10.25)**	1.10 (0.55, 2.20)**		3.28 (1.48, 7.28)*	1.01 (0.51, 1.98)*
Smoking (Past vs Never)		1.68 (0.90, 3.14)	4.21 (1.72, 10.30)		1.45 (0.82, 2.56)	3.70 (1.59, 8.62)
Medications (one more)			1.60 (0.85, 3.01)			1.53 (0.83, 2.83)
Non-Māori Older Adults						
Malnutrition indicator	0.53 (0.38, 0.75)***	0.49 (0.34, 0.70)***	0.53 (0.36, 0.76)***	0.51 (0.31, 0.84)**	0.51 (0.31, 0.86)*	0.50 (0.29, 0.86)*
Sex (M vs F)		1.85 (1.13, 3.03)*	1.95 (1.17, 3.26)*		1.66 (1.05, 2.64)*	1.70 (1.04, 2.79)*
Deprivation (deciles 1–4 vs 8–10)		0.62 (0.35, 1.12)	0.59 (0.32, 1.07)		0.57 (0.33, 1.01)*	0.55 (0.30, 0.99)*
Deprivation (deciles 5–7 vs 8–10)		0.57 (0.34, 0.95)	0.57 (0.34, 0.97)		0.57 (0.35, 0.93)	0.55 (0.33, 0.94)
Smoking (Current vs Never)		2.77 (1.02, 7.57)	3.05 (1.09, 8.57)		3.18 (1.19, 8.49)	3.25 (1.16, 9.14)
Smoking (Past vs Never)		1.53 (0.94, 2.48)	1.52 (0.92, 2.52)		1.38 (0.86, 2.21)	1.43 (0.86, 2.36)
Medications (one more)			1.17 (1.09, 1.25)***			1.18 (1.10, 1.26)***

* p-value <0.05, ** p-value <0.01, ***p-value <0.001; for Deprivation and smoking this is the p-value overall and if significant is indicated next to the first comparison; a. Presence of 1 phenotypic and 1 aetiologic GLIM criteria; b. NZ Deprivation Index (2006) 20; Abbreviations: CI, confidence interval; GLIM, Global Leadership Initiative on Malnutrition; Screen-II, Seniors in the Community: Risk Evaluation for Eating and Nutrition, version II.

Results were similar from the Cox proportional hazard models. Those at higher nutrition risk were much more likely to die at any timepoint in the five years after SCREEN-II (HR (95% CI); 0.52 (0.37, 0.73), P < 0.001 and HR (95% CI); 0.64 (0.47, 0.86), P < 0.01) (Table 4).

**Table 4:** Cox proportional hazards regression models for mortality in five years of follow-up for 142 Māori and 183 non-Māori LiLACS NZ participants identified at nutrition risk

	**SCREEN II (10 points or greater)**			**GLIM malnutrition diagnosis**^**a**^**(no malnutrition vs malnutrition)**		
	**Model A Hazard Ratios (95% CI)**	**Model B Hazard Ratios (95% CI)**	**Model C Hazard Ratios (95% CI)**	**Model A Hazard Ratios (95% CI)**	**Model B Hazard Ratios (95% CI)**	**Model C Hazard Ratios (95% CI)**
Māori Older Adults						
Malnutrition indicator	0.63 (0.45, 0.89)**	0.50 (0.36, 0.71)***	0.52 (0.37, 0.73)***	0.85 (0.52, 1.50)	0.92 (0.55, 1.65)	0.81 (0.48, 1.47)
Age (one year older)		1.14 (1.06, 1.22)***	1.14 (1.06, 1.22)***		1.10 (1.03, 1.17)**	1.11(1.03, 1.19)**
Sex (M vs F)		1.77 (1.13, 2.78)*	1.78 (1.14, 2.80)*		1.71 (1.14, 2.56)*	1.70 (1.09, 2.63)*
Deprivation^b^ (deciles 1–4 vs 8–10)		0.96 (0.47, 1.76)	0.93 (0.46, 1.72)		0.73 (0.37, 1.32)	0.84 (0.42, 1.55)
Deprivation (deciles 5–7 vs 8–10)		1.07 (0.63, 1.76)	1.09 (0.64, 1.78)		0.89 (0.55, 1.40)	1.03 (0.61, 1.69)
Smoking (Current vs Never)		2.71 (1.46, 4.89)**	2.73 (1.47, 4.92)**		2.23 (1.28, 3.79)*	2.44 (1.34, 4.33)**
Smoking (Past vs Never)		1.42 (0.87, 2.32)	1.38 (0.85, 2.26)		1.25 (0.81, 1.96)	1.28 (0.79, 2.09)
Medications (one more)			1.03 (0.97, 1.10)			1.05 (0.99, 1.12)
Non-Māori Older Adults						
Malnutrition indicator	0.58 (0.45, 0.75)***	0.55 (0.42, 0.72)***	0.64 (0.47, 0.86)**	0.54 (0.38, 0.80)**	0.53 (0.37, 0.79)**	0.53 (0.36, 0.80)**
Sex (M vs F)		1.65 (1.12, 2.43)*	1.57 (1.06, 2.33)*		1.56 (1.08, 2.26)*	1.48 (1.00, 2.19)
Deprivation (deciles 1–4 vs 8–10)		0.65 (0.41, 1.02)	0.69 (0.43, 1.08)		0.60 (0.37, 0.93)*	0.59 (0.36, 0.93)*
Deprivation (deciles 5–7 vs 8–10)		0.64 (0.43, 0.96)	0.71 (0.47, 1.06)		0.62 (0.42, 0.91)	0.64 (0.42, 0.95)
Smoking (Current vs Never)		2.41 (1.17, 4.56)*	2.96 (1.42, 5.67)**		2.60 (1.29, 4.83)*	3.13 (1.50, 6.02)**
Smoking (Past vs Never)		1.42 (0.96, 2.11)	1.40 (0.93, 2.09)		1.36 (0.93, 1.99)	1.38 (0.92, 2.06)
Medications (one more)			1.10 (1.05, 1.15)***			1.13 (1.08, 1.18)***

* p-value <0.05, ** p-value <0.01, ***p-value <0.001; for Deprivation and smoking this is the p-value overall and if significant is indicated next to the first comparison; a. Presence of 1 phenotypic and 1 aetiologic GLIM criteria; b. NZ Deprivation Index (2006) (20); Abbreviations: CI, confidence interval; GLIM, Global Leadership Initiative on Malnutrition; Screen-II, Seniors in the Community: Risk Evaluation for Eating and Nutrition, version II.

### Malnutrition diagnosis as a predictor of mortality

A malnutrition diagnosis, as defined by the GLIM criteria, did not significantly predict five-year mortality for Māori (P >0.05) (Table 3). In contrast, malnourished non-Māori participants had greater odds of death within the five year follow up period (Table 3 and 4). This remained significant after adjustment for other predictors of death (OR (95% CI); 0.50 (0.29, 0.86), P < 0.05) (Table 3).

Results for all models did not alter when hs-CRP was substituted for disease burden as the criterion for inflammation. That is, regardless of the measure of inflammation the GLIM criteria were not significantly predictive of five-year mortality for Māori but were for non-Māori. Individual phenotypic criteria were predictive of death for non-Māori but not for Māori, although the significance for non-volitional weight loss for non-Māori was lost when the model was adjusted for co-morbidities (Table 5). Reduced food intake increased the risk of five-year mortality for both Māori (HR (95% CI); 10.77 (4.76, 24.38). P < 0.001), and non-Māori participants (HR (95% CI); 6.69 (2.03, 22.04), P = 0.002).

**Table 5:** Cox proportional hazard regression models for mortality in five years of follow-up for 142 Māori and 183 non-Māori LiLACS NZ participants by individual GLIM criteria

	**Māori**			**Non-Māori**		
	**Model A Odds Ratios (95% CI)**	**Model B Odds Ratios (95% CI)**	**Model C Odds Ratios (95% CI)**	**Model A Odds Ratios (95% CI)**	**Model B Odds Ratios (95% CI)**	**Model C Odds Ratios (95% CI)**
GLIM Phenotypic Factors						
Non-volitional weight loss	1.62 (0.67, 3.88)	1.23 (0.48, 3.13)	1.24 (0.50, 3.12)	2.11 (1.14, 3.94)*	2.18 (1.18, 4.03)*	1.88 (0.95, 3.69)
Low body mass index	1.36 (0.58, 3.17)	1.70 (0.66, 4.35)	2.02 (0.79, 5.17)	1.88 (1.05, 3.37)*	2.28 (1.30, 4.02)**	2.18 (1.20, 3.95)*
Reduced muscle mass	1.71 (0.80, 3.66)	1.46 (0.63, 3.37)	1.58 (0.61, 4.07)	2.07 (1.27, 3.39)**	2.08 (1.26, 3.42)**	2.3 (1.36, 3.87)**
GLIM Aetiologic Factors						
Reduced food intake	14.94 (9.22, 24.19)***	14.06 (6.56, 30.17)***	10.77 (4.76, 24.38)***	3.55 (1.03, 12.20)*	5.08 (1.24, 20.83)*	6.69 (2.03, 22.04)**
Disease burden/inflammation	0.77 (0.34, 1.72)	0.82 (0.38, 1.76)	-	2.71 (0.43, 17.22)	3.05 (0.52, 17.71)	-

Model A, univariate; Model B, adjusted for age (Māori only), sex, deprivation, and smoking status; Model C, further adjusted for comorbidity number and medications. * p-value <0.05, ** p-value <0.01, ***p-value <0.001; Abbreviations: CI, confidence interval; GLIM, Global Leadership Initiative on Malnutrition.

## Discussion

Using the GLIM criterion, we found the prevalence of malnutrition was low for Māori and non-Māori (15% and 19% respectively), despite approximately half of Māori (56%) and non-Māori (46%) being identified with nutrition risk using SCREEN II. Of the phenotypic and aetiologic GLIM criteria, reduced muscle mass was less prevalent for Māori than non-Māori (6% vs 14%). The GLIM consortium acknowledges that reference standards for muscle mass may require adjustment for ethnicity (12, 23). This is particularly pertinent for Māori who have a higher proportion of lean body mass compared to non-Māori, even after adjustment for age, height, and weight (17). These findings highlight the importance of recent recommendations from the GLIM consortium for identifying validated ethnic-specific phenotypic cut-off values (23). Valid body composition data for Māori of advanced age is required to strengthen the reliability of the GLIM criteria for this group.

Our finding of a positive association between greater nutrition risk (SCREENII ≤49) and mortality is consistent with previous studies of older adults (5, 6, 24, 25). In Canada, community-dwelling men (mean age 86.7 years) were found to have a 4% greater risk of death over five years for each 1-point decrease in SCREEN II score (HR (95% CI); 0.96 (0.94, 0.98), P < 0.001) (24). GLIM-diagnosed malnutrition has previously been associated with mortality risk in community dwelling older adults from Belgium, Spain and Hong Kong (14, 15, 16). For the first time, we have shown that GLIM was predictive of five-year mortality in non-Māori adults of advanced age. This association between GLIM diagnosis and death was not observed for Māori, despite higher nutrition risk being predictive of death for Māori. These contrasting findings may in part be explained by the composition of the SCREEN-II tool which places greater emphasis on food intake and access rather than any physical criterion, such as BMI and body composition. This is supported by our finding that phenotypic GLIM criteria were not individually associated with five-year mortality for Māori. Furthermore, low scores in the food intake domain (skipping meals, poor appetite and low intake of fruit, vegetables, and protein foods) of SCREEN-II have previously been associated with higher nutrition risk (4, 7) and with five-year mortality for Māori (7). It is well established that colonisation has adversely altered access to traditional foods, language, and culture; factors that have previously been associated with higher nutrition risk (4, 26, 27).

These findings emphasise the importance of both malnutrition screening and diagnosis for older adults, however, malnutrition diagnosed by GLIM may not be as relevant for Māori in its current form. Targeted nutrition intervention and counselling have been shown to improve body weight and function, while reducing hospital readmissions and healthcare costs for malnourished older adults (28, 29, 30). Health strategies for Māori of advanced age should focus on facilitating engagement in cultural food practices and access to traditional foods. Early intervention and prevention of malnutrition continues to be an important focus of health care in advanced age.

### Strengths and Limitations

The accuracy of BIA can be influenced by hydration status and may overestimate lean mass in very obese patients (31). Despite these limitations, it is a practical and useful tool for estimating body composition in epidemiologic groups (31) and has been used in previous studies of advanced aging (32). The GLIM working group recommends the use of BIA where suitable expertise is available (23). BIA measurements in this study were performed by trained research nurses using standardised BIA protocols (18).

This study applied the GLIM criteria retrospectively and relied on self-reported measures for two criteria (‘reduced food intake' and ‘non-volitional weight loss'). Participant interpretation of questionnaire items, including reduced food intake and weight change, were checked during validation of the SCREEN-II tool (11) and the feasibility phase of LiLACS NZ (33). Culturally appropriate comment was provided by the Rōpū Kaitiaki o tikanga Māori (a governance group to protect the principles of proper conduct for Māori in research) along with Kaumatua and Kuia (male and female Māori elders). This input informed interviewer training to ensure that the purpose of questions was communicated accurately to participants (33).

The minimum data set for retrospective GLIM studies is met in the present study by utilising the objective measures of FFMI and disease burden (13). Furthermore, the prevalence of malnutrition identified in the current study was similar to previous GLIM studies involving older adults where objective measures for ‘reduced food intake' and ‘non-volitional weight loss' were used (14, 15).

## Conclusion

To the best of our knowledge this is the first study to assess malnutrition using the GLIM criteria with indigenous people and those of advanced age. Lower SCREEN-II scores indicative of nutrition risk was a strong predictor of mortality of New Zealand Māori and non-Māori octogenarians, (OR (95% CI); 0.44 (0.27, 0.73), P < 0.01 and OR (95% CI); 0.53 (0.36, 0.76), P < 0.001 respectively). The GLIM criteria identified few participants as malnourished and was not predictive of mortality for Māori. Screening for nutrition risk remains an important tool for clinicians. Our findings concur with the recent advice published by the GLIM consortium, that further work is needed to determine reliable phenotypic criteria for diverse populations (23).
